# Abnormal Stop Band Behavior Induced by Rotational Resonance in Flexural Metamaterial

**DOI:** 10.1038/s41598-018-32597-7

**Published:** 2018-09-24

**Authors:** Sung Won Lee, Joo Hwan Oh

**Affiliations:** School of Mechanical, Aerospace and Nuclear Engineering, Ulsan National Institute of Science and Technology, UNIST-gil 50, Eonyang-eup, Ulju-gun, Ulsan, 44919 Korea

## Abstract

This paper investigates abnormal stop band behavior of resonance-based flexural elastic metamaterials under the rotational resonance motion. Due to the unique physics of flexural waves, we found that the stop band generated by the rotational resonance motion exhibits peculiar behavior which are quite different from general belief – it is shown that the negativity due to the rotational resonance does not provide any stop bands and the stop band generation due to the rotational resonance is governed by totally different band gap condition. To explain the peculiar behavior, a discrete Timoshenko beam model with both effective mass and rotational inertia as independent variables is introduced, and the wave behaviors of resonance-based flexural elastic metamaterial are precisely and fully described. The unique band gap condition, including the peculiar behavior, is derived with numerical validations. We expect our new model can provide a strong background for various flexural elastic metamaterials which can be effectively applied in various vibration devices.

## Introduction

Flexural metamaterials, sub-wavelength periodic structures considering flexural elastic waves, have received much attentions since they are governed by different physics compared to other type of metamaterials. From the frontier researches of Smith^1^, Pendry^2,3^ (in electromagnetics) and Liu *et al*.^4^ (in elastics), various advances have been made for elastic metamaterials exhibiting negative parameters, such as negative density, shear and Young’s modulus^5–18^. As the recent advances in metamaterials have extended our knowledge to various wave phenomena such as negative refraction^17–22^ or super resolution^23–25^, revealing unique physics of flexural metamaterial may open a new way in various vibration problems. Nevertheless, the physics of flexural metamaterials are not fully explained yet due to its unique physics dominated by two different types of deformation – vertical and rotational motions^26^.

**Figure 1:**
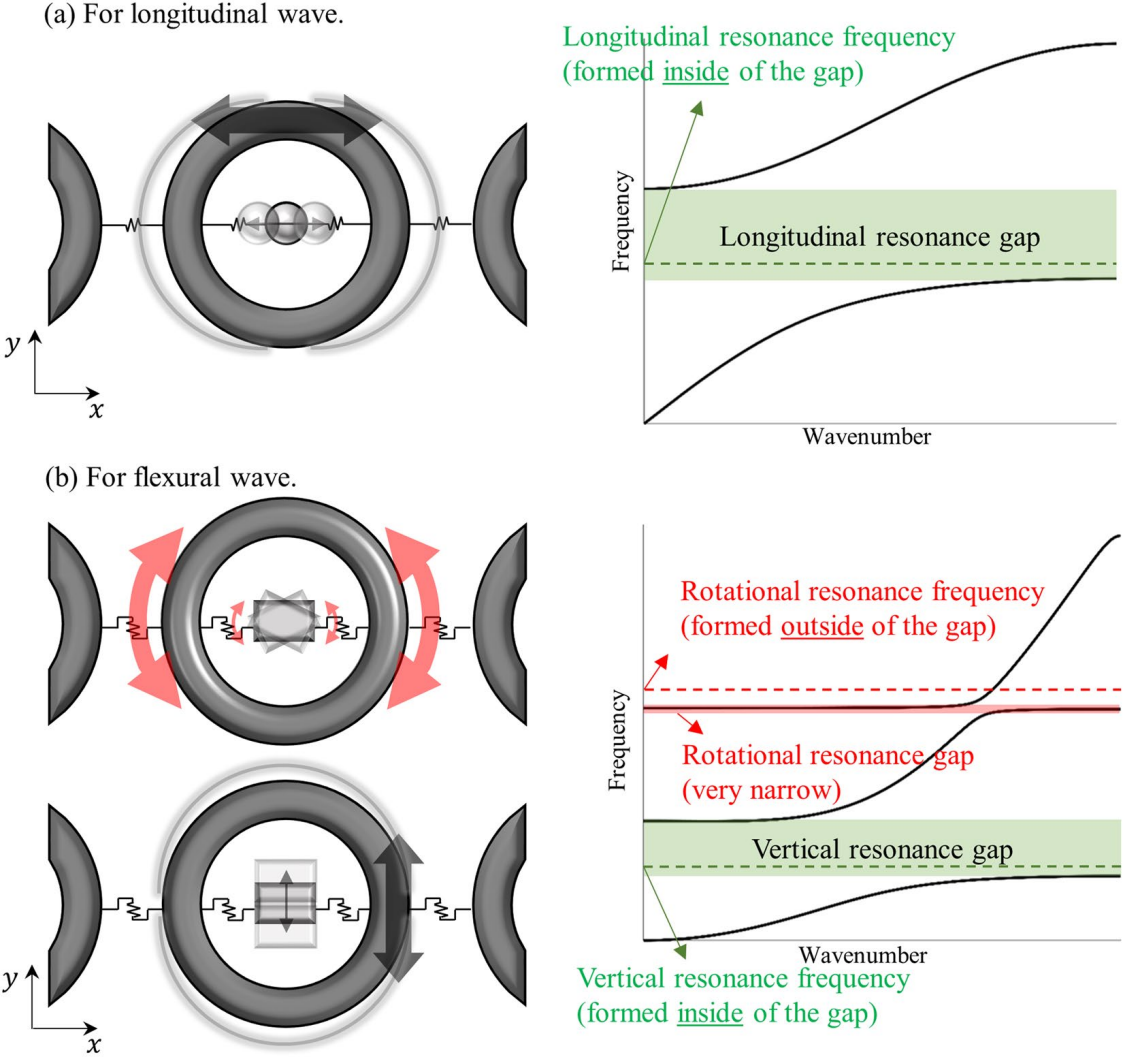
Typical mass-spring systems with internal resonator and dispersion curve for (**a**) longitudinal and (**b**) flexural elastic waves.

To more clearly explain this point, Fig. 1 is prepared with typical dispersion curves of the mass-spring system mimicking elastic metamaterials with internal resonators. If the system is under the longitudinal motion, it has been already known that internal resonance motion provides negative density, which forms a stop band around the internal resonance frequency as in Fig. 1(a). On the other hand, the system’s behavior is totally different if it is under the flexural motion; since the flexural wave is governed by both the vertical and the rotational motion, two kinds of resonance, vertical and rotational one, affect the flexural wave, forming two distinct stop bands. Especially, one can easily find in Fig. 1(b) that the stop band around the rotational resonance frequency exhibits peculiar characteristics that may go beyond the general belief on the resonance-based metamaterials; the stop band is formed outside the actual resonance frequency, and its bandwidth is too narrow. These peculiar characteristics cannot be explained with previous theories on negative density or stiffness, and there has been a need for a new theory of resonance-based flexural metamaterial.

However, previous researches have mainly focused on the vertical resonance motion exhibiting well-known band gap behaviors, not the rotational motion exhibiting peculiar behaviors. Zhu *et al*.^27^ proposed elastic metamaterial shielding flexural waves with negative density. Yu *et al*.^28^ analyzed and confirmed the local resonance band gap in Timoshenko beams by both numerical simulations and experiments. Gusev *et al*.^29^ proposed not only the negative density but also the negative flexural modulus in metaplate with lumped-element approach. Pai *et al*.^30^ proposed the elastic metamaterial which shows both negative density and stiffness by multi-frequencies absorber for flexural waves. Oh *et al*.^31^ proposed the flexural metamaterial which has extremely low stop band, near zero frequency, by realizing zero rotational stiffness. While previous researches provided strong analytic models consisting of Kirchhoff plate with attached mass-springs that can clearly explain the related physics of the vertical resonance phenomena, the physics related to the rotational resonance phenomena has not been clearly explained yet. Although the abnormal stop band behavior due to the rotational resonance can be found in various literatures, the related physics has been still unknown.

In this research, the physics related to the rotational resonance in resonance-based flexural metamaterials are studied. Especially, we found that the stop band generated by the rotational resonance is governed by the totally different condition compared to the generally known theories; although the rotational resonance provides negative parameter as usual, the stop band is not achieved by the negative parameter but by the positive parameter at a certain range. To explain the related physics, we developed a discrete Timoshenko beam model in which an idea of ‘effective rotational inertia’ is introduced in addition to the generally known ‘effective mass’. From the model, effective density and rotational inertia are defined as a function of the vertical and rotational resonance frequencies. Detailed band gap condition for the rotational resonance motion is derived with numerical supports. Finally, as a possible application of the current findings, a new type of frequency filter is shown by combining both the rotational and vertical resonance phenomena.

## Result

### Discrete Timoshenko beam model for the flexural metamaterial

#### Background physics: mass-spring system for flexural wave

**Figure 2:**
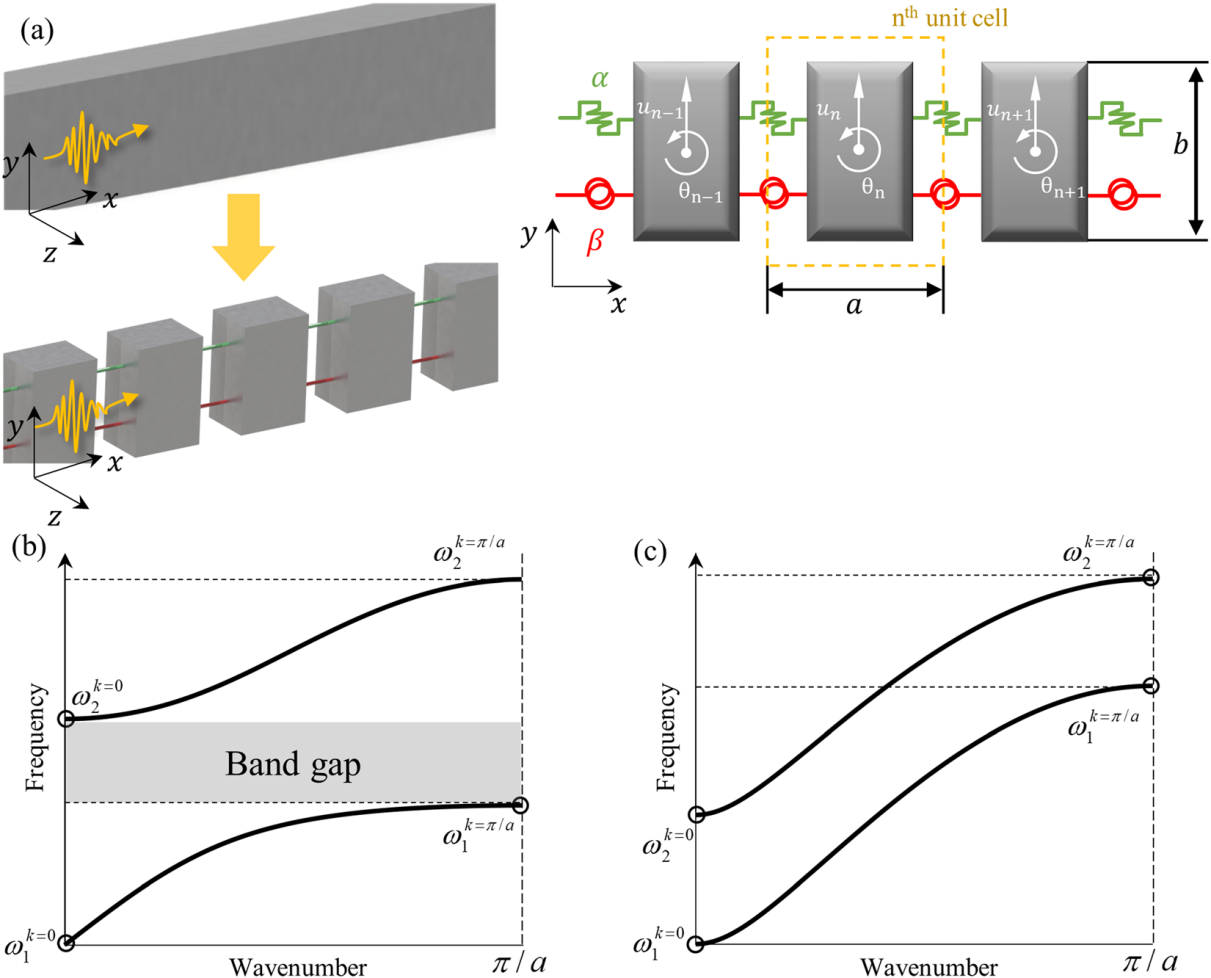
(**a**)Equivalent mass-spring system for general flexural wave, two typical wave dispersion curves of flexural wave if (**b**) *αa*^2^ > 4*β* and (**c**) *αa*^2^ ≤ 4*β*.

As explained previously, since flexural wave is governed by both the vertical and rotational displacements, general mass-spring system used for acoustics or other elastic metamaterials cannot be used. Figure 2(a) shows the mass-spring system for the flexural wave, which is developed from the classical Timoshenko theory^26,31^. Here, two kinds of springs, *α* and *β*, are used to describe the effects of the vertical and rotational displacements, *u*_*n*_ and *θ*_*n*_, respectively. Following the detailed analysis shown in the supplementary material (See Supplementary material for the detailed analytic procedures), the vertical and rotational dynamic equation of the n^th^ unit cell in Fig. 2(a) is derived as^31^:

1a $$-{\omega }^{2}m{u}_{n}=2\alpha \{\cos (ka)-1\}{u}_{n}+i\alpha a\,\sin (ka){\theta }_{n},$$

1b $$-{\omega }^{2}I{\theta }_{n}=-\,i\alpha a\,\sin (ka){u}_{n}+2\beta \{\cos (ka)-1\}{\theta }_{n}-\frac{\alpha {a}^{2}}{2}\{\cos (ka)+1\}{\theta }_{n},$$

which can also be arranged in simple 2 × 2 matrix form as:

2 $$(\begin{array}{cc}{\omega }^{2}m+2\alpha \{\cos (ka)-1\} & i\alpha a\,\sin (ka)\\ -i\alpha a\,\sin (ka) & {\omega }^{2}I+2\beta \{\cos (ka)-1\}-\frac{\alpha {a}^{2}}{2}\{\cos (ka)+1\}\end{array})(\begin{array}{c}{u}_{n}\\ {\theta }_{n}\end{array})=0.$$

To avoid any trivial solution, the determinant of the matrix in Eq. (2) should be zero. This condition yields the wave dispersion equation of the flexural elastic wave as

3 $$\begin{array}{c}[m{\omega }^{2}+2\alpha \{\cos (ka)-1\}][(I{\omega }^{2}+2\beta \{\cos (ka)-1\}-\frac{\alpha {a}^{2}}{2}\{\cos (ka)+1)\}]\\ \,\,\,\,-\,{\{\alpha a\sin (ka)\}}^{2}=0\end{array}$$

Since Eq. (3) is too complicated to be fully solved, we will focus on the frequencies at *k* = 0 and *k* = *π*/*a*, which corresponds to the starting and terminating frequencies of each dispersion branch in the irreducible Brillouin zone^32^. Substituting *k* = 0 and *k* = *π*/*a* to Eq. (3) yields

4a $${\rm{at}}\,k=0:{\omega }_{1}^{k=0}=0\,{\rm{and}}\,{\omega }_{2}^{k=0}=\sqrt{\alpha {a}^{2}/I},$$

4b $${\rm{at}}\,k=\pi /a:{\omega }_{1}^{k=\pi /a}=\sqrt{4\beta /I}\,{\rm{and}}\,{\omega }_{2}^{k=\pi /a}=\sqrt{4\alpha /m}.$$

Considering that there is no mode coupling (between flexural and other wave modes) and the system is 1-Dimensional system, the corresponding wave dispersion branches should monotonically increase or decrease without any branch-overlapping. Thus, as shown in Fig. 2(b,c), it can be expected that the first flexural wave’s dispersion branch lies from ${\omega }_{1}^{k=0}=0$ to ${\omega }_{1}^{k=\pi /a}=\sqrt{4\beta /I}$, while that for the second branch is from ${\omega }_{2}^{k=0}=\sqrt{\alpha {a}^{2}/I}$ to ${\omega }_{2}^{k=\pi /a}=\sqrt{4\alpha /m}$. Therefore, if *αa*^2^ > 4*β*, there should be a band gap from $\sqrt{\alpha {a}^{2}/I}$ to $\sqrt{4\beta /I}$ as shown in Fig. 2(b). (As can be seen in the supplementary material, *αa*^2^ is generally much larger than 4*β*). Based on these results, detailed physics of the resonance-based flexural metamaterial will be studied.

#### Discrete Timoshenko beam model for resonance-based flexural metamaterial

**Figure 3:**
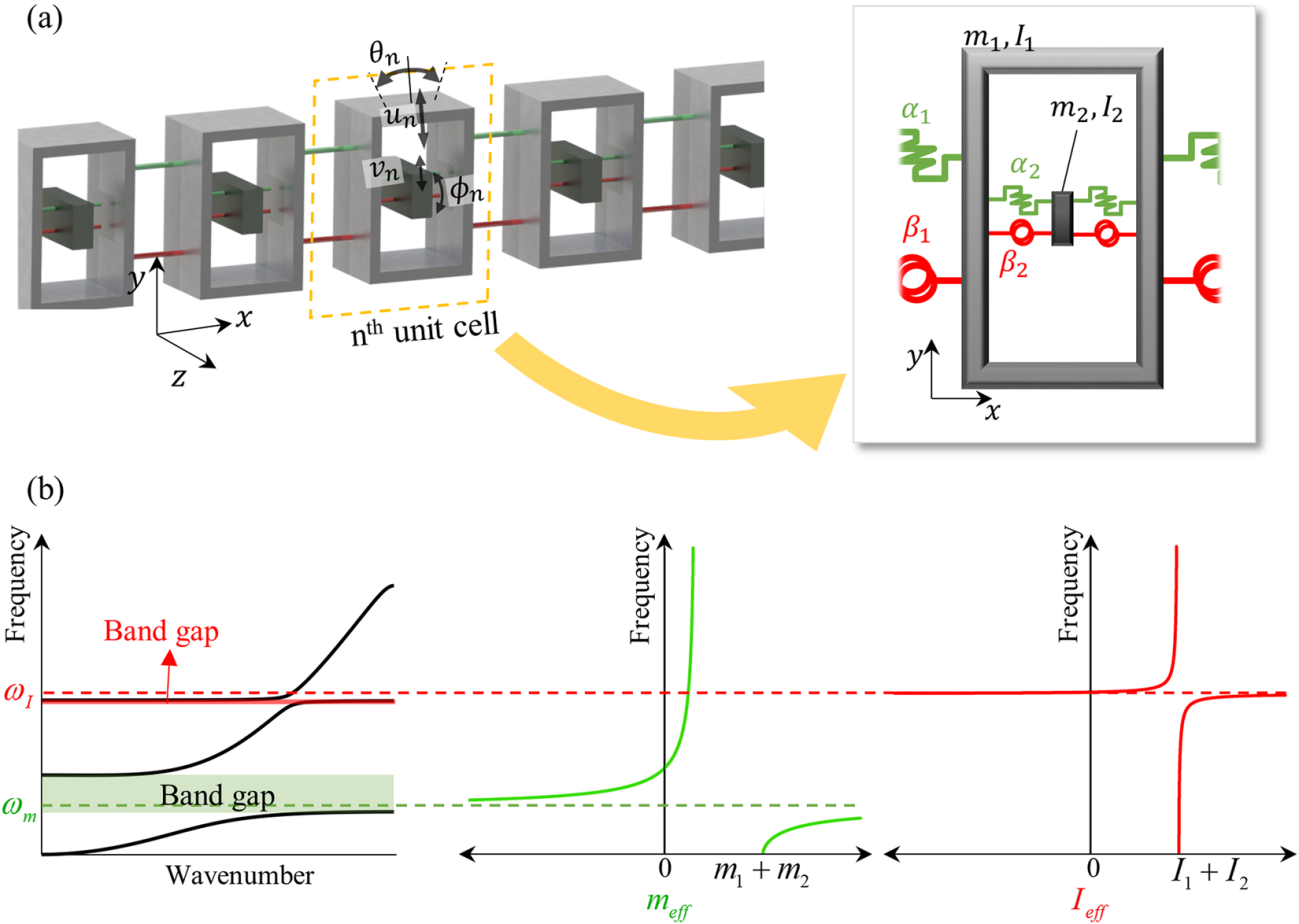
(**a**) Equivalent mass-spring system for flexural elastic metamaterial with internal resonator, (**b**) plots of the dispersion curve and effective parameters of the mass-spring system shown in (**a**).

Now, let us focus on the flexural metamaterial with inner resonator, whose mass-spring system is shown in Fig. 3(a). To avoid any complexity such as the band crossing, we will consider the frequencies below the homogenization limit, i.e., only the first wave dispersion curve below the frequency ${\omega }_{1}^{k=\pi /a}=\sqrt{4\beta /I}$ is studied. Based on the mass-spring system shown in Fig. 3(a), the vertical and rotational dynamic equations of the n^th^ outer mass can be calculated as (the detailed process can be found in the supplementary material.

5a $$-{\omega }^{2}({m}_{1}+\frac{2{\alpha }_{2}{m}_{2}}{2{\alpha }_{2}-{\omega }^{2}{m}_{2}}){u}_{n}=2{\alpha }_{1}\{\cos (k{a}_{1})-1\}{u}_{n}+i{\alpha }_{1}{a}_{1}\,\sin (k{a}_{1}){\theta }_{n},$$

5b $$\begin{array}{rcl}-{\omega }^{2}({I}_{1}+\frac{(2{\beta }_{2}+{\alpha }_{2}{a}_{2}^{2}/2){I}_{2}}{2{\beta }_{2}+{\alpha }_{2}{a}_{2}^{2}/2-{\omega }^{2}{I}_{2}}){\theta }_{n} & = & -\,i{\alpha }_{1}{a}_{1}\,\sin (k{a}_{1}){u}_{n}\\  &  & \begin{array}{c}+\,2{\beta }_{1}\{\cos (k{a}_{1})-1\}{\theta }_{n}\\ -\,\frac{{\alpha }_{1}{a}_{1}^{2}}{2}\{\cos (k{a}_{1})+1\}{\theta }_{n}.\end{array}\end{array}$$

Comparing Eqs (1a,b) and (5a,b), one can find that Eq. (5a,b) (defined for mass-spring system with internal resonator shown in Fig. 3(a)) can be converted to Eqs (1a,b) (defined for mass-spring system in Fig. 2(a)) if the effective mass and inertia, *m*_*eff*_ and *I*_*eff*_, are defined as

6ab $${m}_{eff}={m}_{1}+\frac{2{\alpha }_{2}{m}_{2}}{2{\alpha }_{2}-{\omega }^{2}{m}_{2}},\,\,{I}_{eff}={I}_{1}+\frac{(2{\beta }_{2}+{\alpha }_{2}{a}_{2}^{2}/2){I}_{2}}{2{\beta }_{2}+{\alpha }_{2}{a}_{2}^{2}/2-{\omega }^{2}{I}_{2}}.$$

Here, let’s define two resonance frequencies as ${\omega }_{m}^{2}=2{\alpha }_{2}/{m}_{2}$ and ${\omega }_{I}^{2}=(2{\beta }_{2}+{\alpha }_{2}{a}_{2}^{2}/2)/{I}_{2}$, which correspond to the vertical and rotational resonance frequency, respectively. With *ω*_*m*_ and *ω*_*I*_, Eq. (6) can be re-written as

7ab $${m}_{eff}={m}_{1}+\frac{{\omega }_{m}^{2}{m}_{2}}{{\omega }_{m}^{2}-{\omega }^{2}},\,\,\,{I}_{eff}={I}_{1}+\frac{{\omega }_{I}^{2}{I}_{2}}{{\omega }_{I}^{2}-{\omega }^{2}}.$$

It should be noted that the effective inertia *I*_*eff*_ in Eq. (7b) is defined for the flexural motion, which should be distinguished from the rotational inertia defined for the torsional motion. Although both parameters are affected by the rotational resonance motion, the axis of rotation for the flexural wave is perpendicular to the wave propagating direction (in Fig. 3(a), the axis of rotation is *z*-axis) while the axis of rotation for the torsional wave is parallel to the wave propagating direction. As experimentally shown from previous research^33^, the physics related to the effective rotational inertia for torsional motion are almost same as the generally known physics for the effective mass. However, the physics related to the effective rotational inertia for flexural motion is different from the well-known physics as explained in Fig. 1(b).

Figure 3(b) shows the typical wave dispersion curve of the mass-spring system in Fig. 3(a), with the plots of *m*_*eff*_ and *I*_*eff*_. From Fig. 3(b) and Eq. (7), the following three major findings can be observed;

1. Each internal resonance frequency only affects on the corresponding effective parameter, respectively. The vertical resonance frequency *ω*_*m*_ only affects the effective mass *m*_*eff*_, while the rotational resonance frequency *ω*_*I*_ only affects the effective inertia *I*_*eff*_ (However, *ω*_*m*_ and *ω*_*I*_ are not totally independent since both are function of vertical spring coefficient *α*_2_).
2. Similar to the well-known findings on the negative density^6,15^, *m*_*eff*_ and *I*_*eff*_ can be an infinite or negative value around the internal resonance frequency *ω*_*m*_ and *ω*_*I*_, respectively.
3. Unlike the band gap generated by *ω*_*m*_, the band gap generated by *ω*_*I*_ is not identical to the frequency range where *I*_*eff*_ is negative, i.e., negative *I*_*eff*_ does not produce band gap. Furthermore, the rotational resonance frequency *ω*_*I*_ is located outside the band gap, while the vertical resonance frequency *ω*_*m*_ is inside the band gap.

The third finding indicates that the band gap condition for the rotational inertia is different from the condition for the mass where negative mass provides band gap. Since this point is closely related to the peculiar behavior of the flexural metamaterial, it will be intensively studied in the next section.

#### Band gap condition for in flexural elastic metamaterial

To investigate the unique band-gap condition in flexural metamaterial, Eq. (5) is re-arranged into simple 2 × 2 matrix form as:

8 $$(\begin{array}{cc}{\omega }^{2}{m}_{eff}+2{\alpha }_{1}\{\cos (k{a}_{1})-1\} & i{\alpha }_{1}{a}_{1}\,\sin (k{a}_{1})\\ -i{\alpha }_{1}{a}_{1}\,\sin (k{a}_{1}) & {\omega }^{2}{I}_{eff}+2{\beta }_{1}\{\cos (k{a}_{1})-1\}-\frac{{\alpha }_{1}{{a}_{1}}^{2}}{2}\{\cos (k{a}_{1})+1\}\end{array})(\begin{array}{c}{u}_{n}\\ {\theta }_{n}\end{array})=0.$$

Again, the determinant of the matrix in Eq. (8) should be zero for non-trivial condition. Therefore, the wave dispersion equation can be derived as

9 $$\begin{array}{c}[{m}_{eff}{\omega }^{2}+2{\alpha }_{1}(\cos (k{a}_{1})-1]\,[{I}_{eff}{\omega }^{2}+2{\beta }_{1}\{\cos (k{a}_{1})-1\}-\frac{{\alpha }_{1}{a}_{1}^{2}}{2}\{\cos (k{a}_{1})+1\}]\\ \,\,\,-\,{[{\alpha }_{1}{a}_{1}\sin (k{a}_{1})]}^{2}=0.\end{array}$$

Considering that both *m*_*eff*_ and *I*_*eff*_ are functions of frequency *ω*, solving Eq. (9) is too complicated and may not provide any good insight for the band gap condition. Thus, as done previously, the frequencies corresponding to *k* = 0 and *k* = *π*/*a*_1_ will be focused. Substituting *k* = 0 and *k* = *π*/*a*_1_ into Eq. (9) yields

10a $${\rm{at}}\,k=0:{m}_{eff}{\omega }^{2}({I}_{eff}{\omega }^{2}-{\alpha }_{1}{a}_{1}^{2})=0,$$

10b $${\rm{at}}\,k=\pi /{a}_{1}:({m}_{eff}{w}^{2}-4{\alpha }_{1})({I}_{eff}{w}^{2}-4{\beta }_{1})=0.$$

Based on Eq. (10), the band gap condition around the vertical and rotational resonance frequencies, *ω*_*m*_ and *ω*_*I*_, will be studied. Since *ω*_*m*_ and *ω*_*I*_ independently affect *m*_*eff*_ and *I*_*eff*_ (by the first finding), each resonance will be considered separately.

Band gap condition for the vertical resonance: First, assume that the internal resonator exhibits the vertical resonance motion only, i.e., only *ω*_*m*_ exists. In this case, *m*_*eff*_ significantly alters from negative infinite to positive infinite value (by the second finding), while *I*_*eff*_ = *I*_*o*_ = *I*_1_ + *I*_2_ is constant. Thus, the frequencies corresponding to *k* = 0 and *k* = *π*/*a*_1_ can be solved from Eq. (10) as

11a $${\rm{at}}\,k=0:\{\begin{array}{c}{\omega }_{1m}^{k=0}=0\\ {\omega }_{2m}^{k=0}\,\,{\rm{where}}\,\,{m}_{eff}={m}_{1}+\frac{{\omega }_{m}^{2}{m}_{2}}{{\omega }_{m}^{2}-{({\omega }_{2m}^{k=0})}^{2}}=0\\ {\omega }_{3m}^{k=0}=\sqrt{{\alpha }_{1}{{a}_{1}}^{2}/{I}_{o}}\end{array}$$

11b $${\rm{at}}\,k=\pi /{a}_{1}:\{\begin{array}{c}{\omega }_{1m}^{k=\pi /{a}_{1}}\,{\rm{where}}\,{m}_{eff}{({\omega }_{1m}^{k=\pi /{a}_{1}})}^{2}=({m}_{1}+\frac{{\omega }_{m}^{2}{m}_{2}}{{\omega }_{m}^{2}-{({\omega }_{1m}^{k=\pi /{a}_{1}})}^{2}}){({\omega }_{1m}^{k=\pi /{a}_{1}})}^{2}=4{\alpha }_{1}\\ {\omega }_{2m}^{k=\pi /{a}_{1}}=\sqrt{4{\beta }_{1}/{I}_{o}}\\ {\omega }_{3m}^{k=\pi /{a}_{1}} \sim \sqrt{4{\alpha }_{1}/{m}_{1}}\end{array}$$

**Figure 4:**
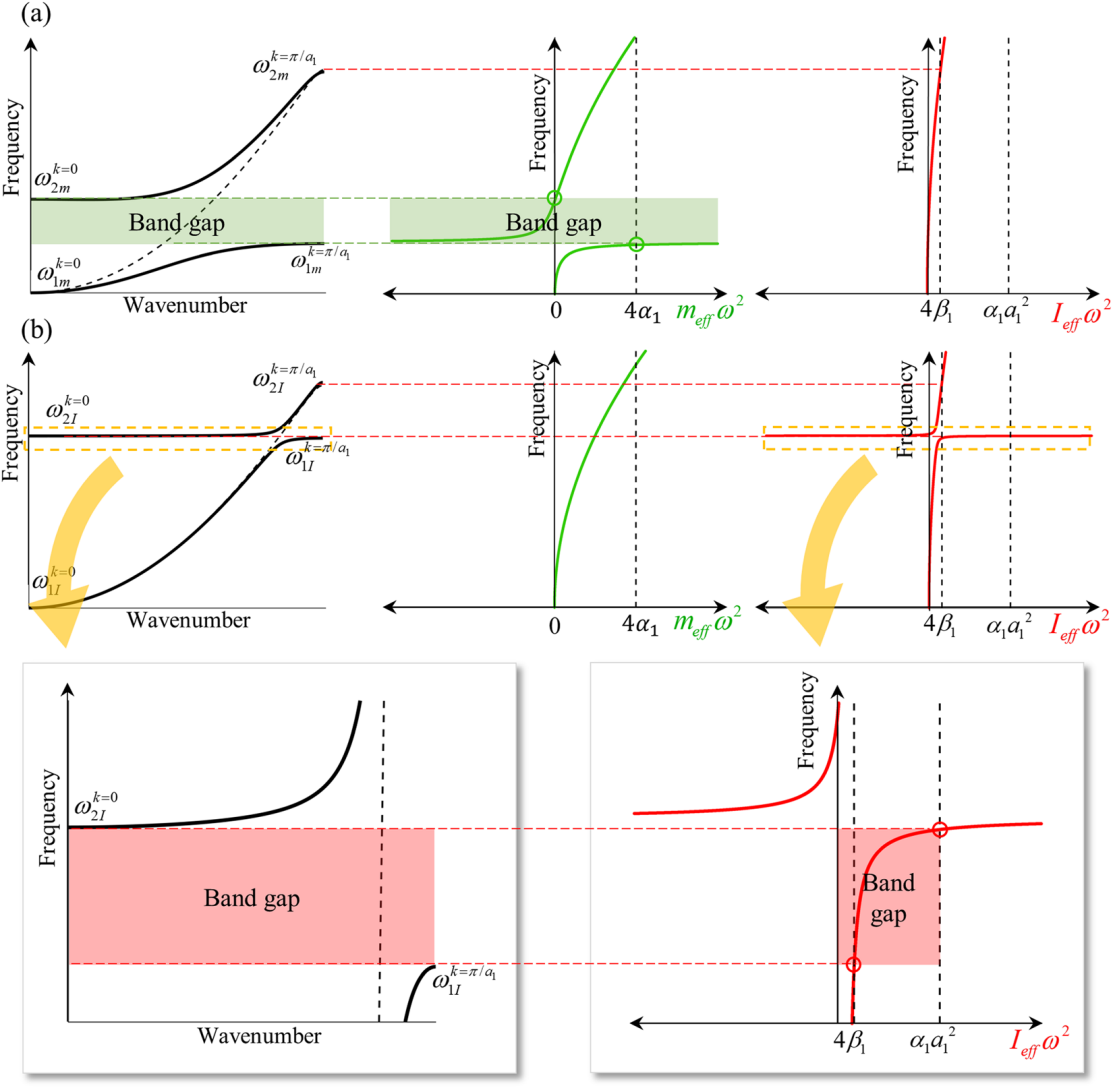
Plots of wave dispersion curve, effective mass and effective rotational inertia if the internal resonator exhibits (**a**) vertical resonance only and (**b**) rotational resonance only.

Comparing Eq. (11) with Eq. (4), one can easily find that ${\omega }_{2m}^{k=0}$ and ${\omega }_{1m}^{k=\pi /{a}_{1}}$ are newly considered since *m*_*eff*_ is not a constant value anymore. Figure 4(a) shows the corresponding wave dispersion curve and effective parameters. Note that ${\omega }_{3m}^{k=0}$ and ${\omega }_{3m}^{k=\pi /{a}_{1}}$ are not plotted since we are focusing on the frequencies below the homogenization limit, i.e., below $\omega =\sqrt{4{\beta }_{1}/{I}_{0}}$. From Fig. 4(a), it can be clearly observed that the vertical resonance creates a new band gap from ${\omega }_{1m}^{k=\pi /{a}_{1}}$ to ${\omega }_{2m}^{k=0}$. This indicates that the band gap is generated if

12 $$4{\alpha }_{1} < {m}_{eff}{\omega }^{2} < {\rm{\infty }}\,({\rm{B}}{\rm{r}}{\rm{a}}{\rm{g}}{\rm{g}}\,{\rm{g}}{\rm{a}}{\rm{p}})\,{\rm{o}}{\rm{r}}\,-\,{\rm{\infty }} < {m}_{eff}{\omega }^{2} < 0\,({\rm{N}}{\rm{e}}{\rm{g}}{\rm{a}}{\rm{t}}{\rm{i}}{\rm{v}}{\rm{e}}\,{\rm{d}}{\rm{e}}{\rm{n}}{\rm{s}}{\rm{i}}{\rm{t}}{\rm{y}}).$$

One can find that the condition shown in Eq. (12) is same as those of the negative density phenomena studied previously^7^. In fact, the physics related to the vertical resonance is same as the well-known physics of negative density. Obviously, this frequency range includes the negative *m*_*eff*_ region and the vertical resonance frequency *ω*_*m*_, as in Fig. 4(a).

Band gap condition for the rotational resonance at *ω*_I_: Now, let us focus on the band gap generated by the rotational resonance, which exhibits the peculiar behaviors. As done previously, assume that the internal resonator exhibits only the rotational resonance motion so that only *ω*_*I*_ exists. Accordingly, *m*_*eff*_ = *m*_*o*_ = *m*_1_ + *m*_2_ while *I*_*eff*_ varies from negative infinite to positive infinite value. With these conditions, the frequencies at *k* = 0 and *k* = *π/a*_*1*_ are evaluated as

13a $${\rm{at}}\,k=0:\{\begin{array}{c}{\omega }_{1I}^{k=0}=0\\ {\omega }_{2I}^{k=0}\,{\rm{where}}\,{I}_{eff}{({\omega }_{2I}^{k=0})}^{2}=({I}_{1}+\frac{{\omega }_{I}^{2}{I}_{2}}{{\omega }_{I}^{2}-{({\omega }_{2I}^{k=0})}^{2}}){({\omega }_{2I}^{k=0})}^{2}={\alpha }_{1}{a}_{1}^{2}\\ {\omega }_{3I}^{k=0} \sim \sqrt{{\alpha }_{1}{a}_{1}^{2}/{I}_{1}}\end{array}.$$

13b $${\rm{at}}\,k=\pi /{a}_{1}:\{\begin{array}{c}{\omega }_{1I}^{k=\pi /{a}_{1}}\,{\rm{where}}\,{I}_{eff}{({\omega }_{1I}^{k=\pi /{a}_{1}})}^{2}=({I}_{1}+\frac{{\omega }_{I}^{2}{I}_{2}}{{\omega }_{I}^{2}-{({\omega }_{1I}^{k=\pi /{a}_{1}})}^{2}}){({\omega }_{1I}^{k=\pi /{a}_{1}})}^{2}=4{\beta }_{1}\\ {\omega }_{2I}^{k=\pi /{a}_{1}} \sim \sqrt{4{\beta }_{1}/{I}_{1}}\\ {\omega }_{3I}^{k=\pi /{a}_{1}}=\sqrt{4{\alpha }_{1}/{m}_{o}}\end{array}.$$

Figure 4(b) shows the wave dispersion curve and effective parameters when the rotational resonance takes place. Again, ${\omega }_{3I}^{k=0}$ and ${\omega }_{3I}^{k=\pi /{a}_{1}}$ are not considered due to the homogenization limit. Due to the rotational resonance, *I*_*eff*_ can have various values from negative infinite to positive infinite. Therefore, ${\omega }_{2I}^{k=0}$ and ${\omega }_{1I}^{k=\pi /{a}_{1}}$ are newly considered, and a new band gap from ${\omega }_{1I}^{k=\pi /{a}_{1}}$ to ${\omega }_{2I}^{k=0}$ is formed.

From Fig. 4(b), one can clearly see that the frequency range of the band gap does not cover the frequency range of the negative *I*_*eff*_. In fact, Eq. (13) suggests that the band gap is generated if the value of the effective inertia term *I*_*eff*_ *ω*^2^ lies between 4*β*_1_ and ${\alpha }_{1}{a}_{1}^{2}$ as seen in Fig. 4(b). This indicates that negativity in the rotational inertia does not provide band gap, unlike density or mass where negativity directly provides the band gap. This obviously explains why the band gap generated by the rotational resonance does not include the rotational resonance frequency as in Fig. 4(b); the stop band is generated where *I*_*eff* _*ω*^2^ lies between 4*β*_1_ and ${\alpha }_{1}{a}_{1}^{2}$ while the rotational resonance frequency belongs to the infinite value of *I*_*eff*_. Also, the band gap condition also explains why the rotation-induced band gap is very narrow. The frequency range at which *I*_*eff*_ *ω*^2^ is between 4*β*_1_ and ${\alpha }_{1}{a}_{1}^{2}$ should be very narrow since *I*_*eff*_ rapidly varies around the rotational resonance frequency *ω*_*I*_.

Here, we considered the case of ${\alpha }_{1}{a}_{1}^{2} > 4{\beta }_{1}$, which is the usual case as explained previously. If ${\alpha }_{1}{a}_{1}^{2}\le 4{\beta }_{1}$, however, the rotational resonance would not provide any band gap since ${\omega }_{1I}^{k=\pi /{a}_{1}}$ is higher than ${\omega }_{2I}^{k=0}$. At this condition, there are two modes of flexural elastic wave at the frequencies between ${\omega }_{2I}^{k=0}$ and ${\omega }_{1I}^{k=\pi /{a}_{1}}$, where the former is the rotation-dominant wave and the latter is the vertical motion-dominant wave^26,31^. As a result, it can be summarized that band gap is achieved if

14a $$4{\beta }_{1} < {I}_{eff}{\omega }^{2} < {\alpha }_{1}{a}_{1}^{2}\,({\rm{i}}{\rm{f}}\,{\alpha }_{1}{a}_{1}^{2} > 4{\beta }_{1}),$$

14b $${\rm{N}}{\rm{o}}\,{\rm{b}}{\rm{a}}{\rm{n}}{\rm{d}}\,{\rm{g}}{\rm{a}}{\rm{p}}\,({\rm{i}}{\rm{f}}\,{\alpha }_{1}{a}_{1}^{2}\le 4{\beta }_{1}).$$

which are totally different conditions from the well-known band gap condition.

### Numerical Validations

#### Validation with dispersion curves

**Figure 5:**
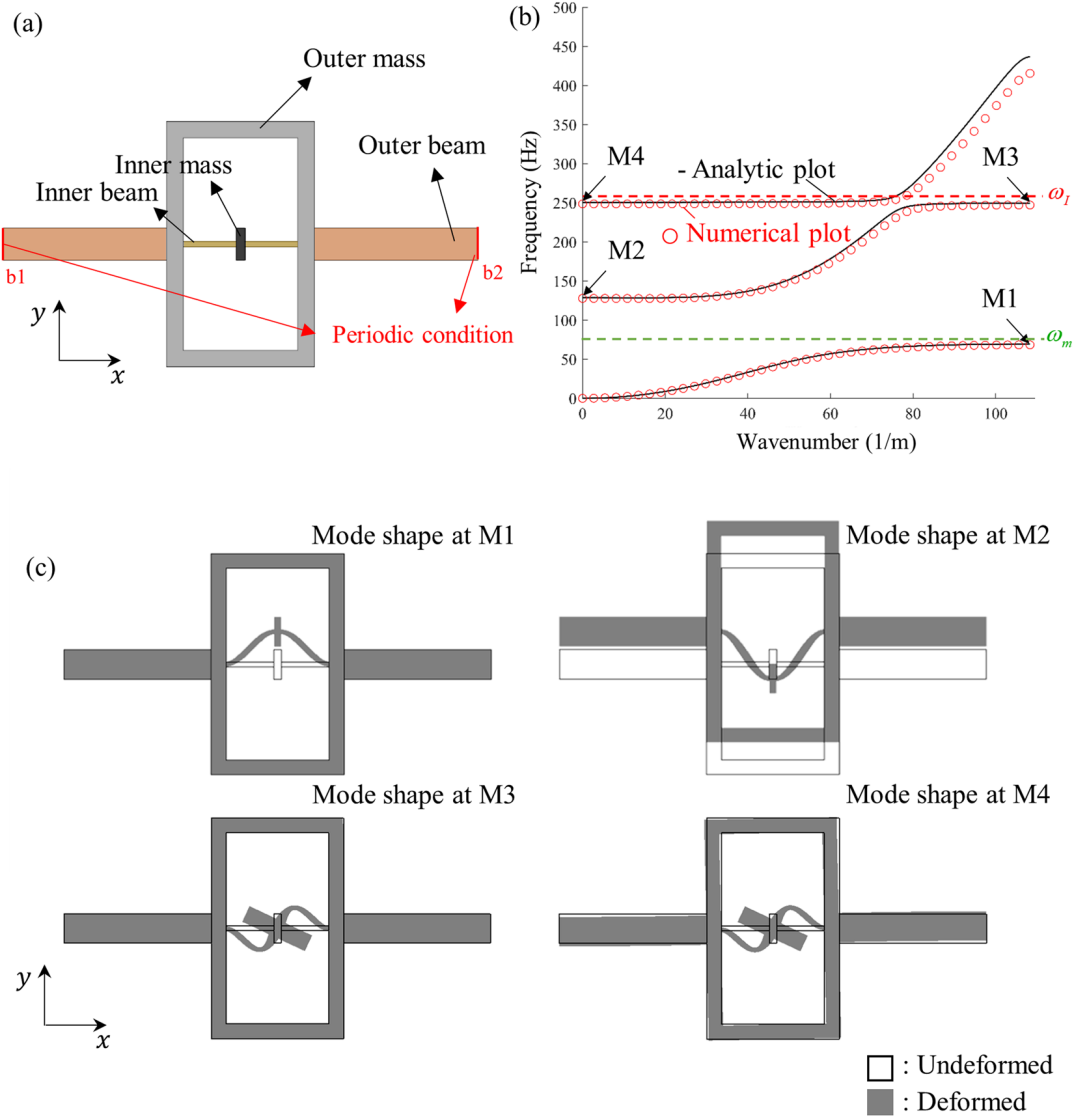
(**a**) Unit cell of the metamaterial considered for the numerical investigations. (**b**) Plots of numerically and analytically calculated wave dispersion curve, (**c**) mode shapes of the unit cell at the points M1, M2, M3, and M4 in (**b**). Note that only flexural mode branches are plotted.

To validate the analytic investigations on resonance based flexural metamaterial, numerical simulations are carried out. First, the wave dispersion curve is calculated to validate the analytical investigation. For the validation, the metamaterial unit cell shown in Fig. 5(a) is considered. The detailed geometric and material parameters of the unit cell are given in our supplementary material. Simulation with the inner beam and mass only showed that the vertical and rotational resonance frequencies are *ω*_*m*_ = 71.18 Hz and *ω*_*I*_ = 250.76 Hz, respectively. Also, it was shown that the unit cell’s resonance, which can highly affect the wave transmission characteristics as shown in ref.^34^, does not exist at the frequency below 300 Hz. With the unit cell shown in Fig. 5(a), the wave dispersion curve is numerically and analytically calculated. For the analytic calculation, the equivalent coefficients, such as *m*_1_, *I*_1_, *α*_1_, and *β*_1_, are calculated by using the classical vibration theory^35^; the detailed values can be found in the supplementary material.

Figure 5(b) compares analytically and numerically calculated dispersion curves. Very good agreements can be observed between two results, validating our theoretical investigations. Obviously, the band gaps are not due to the negative stiffness effect since only the positive stiffness values were considered in the analytic calculation. Two distinct band gaps can be observed around the vertical resonance frequency (68.57 ~ 126.38 Hz) and the rotational resonance frequency (246.90 ~ 247.95 Hz). As predicted from the analytical investigation, the band gap near the rotational resonance frequency is much narrower than the band gap around the vertical resonance frequency. Also, the vertical resonance frequency (71.18 Hz) is inside the band gap (68.57 ~ 126.38 Hz) while the rotational resonance frequency (250.76 Hz) is outside the band gap (246.90 ~ 247.95 Hz), which agrees with our analytic theory.

Figure 5(c) plots the mode shapes at the lower and upper edge frequencies of two band gaps. From Fig. 5(c), one can clearly figure out that the first band gap formed at 68.57 ~ 126.38 Hz is originated from the vertical resonance of the internal resonator, while the second band gap formed at 246.90 ~ 247.95 Hz, is originated from the rotational resonance. It is interesting that the mode shapes at the first band gap’s lower and upper edge frequencies, M1 and M2, are out-of-phase while those at the second band gap’s lower and upper edge frequencies, M3 and M4, are in-phase. (Here, M3 and M4 are not on a same branch – M4 corresponds to the cutoff frequency where the high-order flexural wave branch starts). This can be explained from the classical vibration theory that the mode shapes at the frequencies lower and higher than the internal resonance frequency show 180° phase difference^35^. Since the first band gap includes the vertical resonance frequency inside, the mode shape at M1 and M2 show out-of-phase motion. On the other hand, the in-phase motion at M3 and M4 suggests that the corresponding resonance frequency, the rotational resonance frequency, is at the outside of the second band gap. This also supports our findings that the band gap generated by the rotational resonance does not include the rotational resonance frequency.

#### Validation with wave transmission simulations

**Figure 6:**
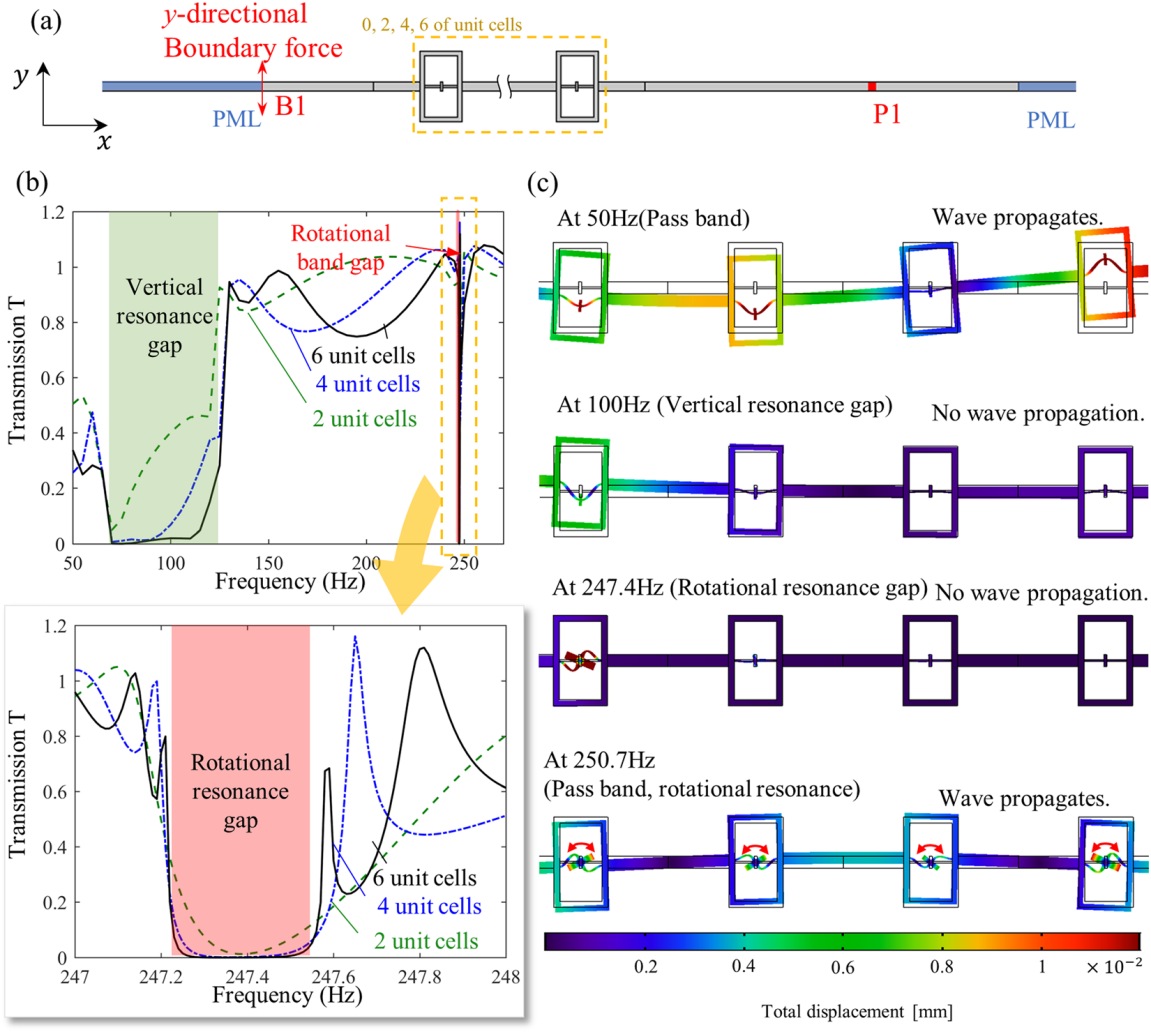
(**a**) A finite element modeling to measure the flexural wave transmission, (**b**) transmission at various frequencies, (**c**) wave simulation results at various frequencies.

In addition to the wave dispersion curve, the wave transmission simulations are carried out to study the detailed wave phenomena for finite metamaterial layers. Figure 6(a) shows the simulation setting for the flexural wave propagation with a finite metamaterial layer consisting of 2, 4, and 6 unit cells. Here, the same unit cell shown in Fig. 5(a) is used. The simulation process is described in the Method section. After all, the transmission T for various frequency, 50 to 270 Hz, is calculated by the described procedure. Note that the transmission T may exceed 1 due to its definition, as explained in the Method section.

Figure 6(b) shows the transmission T measured by the above method for metamaterial layers consisting of 2, 4, and 6 unit cells. Extremely low transmission is measured at the frequencies from 69 ~ 125 Hz and 247.3 ~ 247.5 Hz, which corresponds to the first and second band gap in the dispersion curve shown in Fig. 5(b). Considering that the resonance frequencies are 71.18 Hz and 250.76 Hz, the analytic finding can be validated here; the vertical resonance frequency belongs to the stop band, while the rotational resonance frequency belongs to the pass band. It should be noted that the low transmission at these frequency bands can be observed regardless of the number of unit cells, which indicates that the low transmission is not because of the Fabry-Perot anti-resonance. Also, although the second band gap at 247.3 ~ 247.5 Hz (originated from the rotational resonance) is extremely narrow, it can be observed that very low transmission is well-formed with two unit cells.

Figure 6(c) shows the deformed configuration of the metamaterial layer consisting of 4 unit cells at various frequencies. The flexural elastic wave propagates inside the metamaterial layer, which implies that the frequency belongs to the pass band. On the other hand, at 100 Hz which belongs to the first band gap, it can be seen that the wave cannot propagate through due to the vertical resonance motion of the first unit cell. In the same manner, the flexural wave at 247.4 Hz (which belongs to the second band gap) cannot propagate through because the first unit cell exhibits rotational resonance motion. These two results clearly show that the first and second band gap is originated from the vertical and rotational resonance, respectively, which is identical to the previous analytic finding that each resonance motion generates independent stop band.

In addition, it was shown that the rotational resonance frequency *ω*_*I*_ (250.7 Hz) belongs to the pass band in the previous analytic investigation. To check this point, the wave simulation result at the rotational resonance frequency is shown in Fig. 6(c). Although the internal resonators exhibit large rotational motion, it can be seen that not only the first unit cell but also all the other unit cells exhibit rotational motion. This clearly indicates that *ω*_*I*_ belongs to the pass band.

### The band gap overlapping for frequency filtering application

From the results shown above, one may argue that the band gap generated by the rotational inertia is almost meaningless since the band gap is too narrow to be applied in actual applications such as vibration shielding. However, by extending the current theory, it is possible to form very narrow pass band inside a broad band gap which is highly preferred in the frequency filtering applications. As a possible application that can be inferred from the current research, a frequency filtering device will be introduced here.

**Figure 7:**
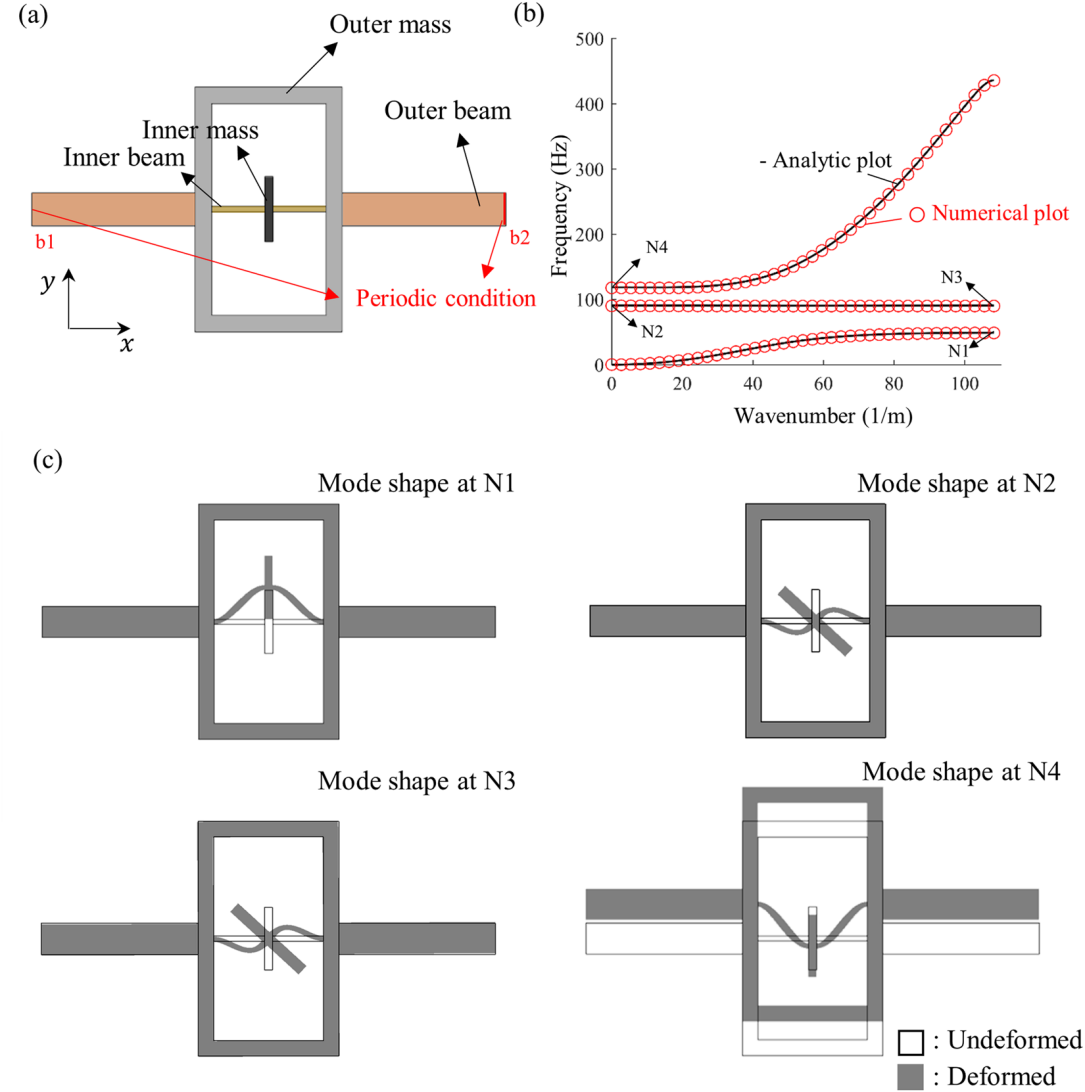
(**a**) Unit cell of the metamaterial for the frequency filtering, (**b**) plots of numerically and analytically calculated wave dispersion curve, (**c**) mode shapes of the unit cell at the points N1, N2, N3 and N4 in (b). Note that only flexural mode branches are plotted.

Consider a flexural metamaterial whose internal resonator exhibits vertical resonance motion at *ω*_*m*_. Due to the vertical resonance motion, a band gap is generated from ${\omega }_{1m}^{k=\pi /{a}_{1}}$ to ${\omega }_{2m}^{k=0}$ (the definition of ${\omega }_{1m}^{k=\pi /{a}_{1}}$ and ${\omega }_{2m}^{k=0}$ can be found in Eq. (11)). Now, assume that the internal resonator also exhibits rotational resonance motion at *ω*_*I*_ while *ω*_*I*_ is inside the band gap from ${\omega }_{1m}^{k=\pi /{a}_{1}}$ to ${\omega }_{2m}^{k=0}$. As can be seen in Fig. 7(b), there should be an additional dispersion branch between ${\omega }_{1I}^{k=\pi /{a}_{1}}$ and ${\omega }_{2I}^{k=0}$ (the definition of ${\omega }_{1I}^{k=\pi /{a}_{1}}$ and ${\omega }_{2I}^{k=0}$ are given in Eq. (13)). Because the frequency range from ${\omega }_{1I}^{k=\pi /{a}_{1}}$ to ${\omega }_{2I}^{k=0}$ is very narrow, the new dispersion branch will provide very narrow rotation-induced pass band inside the broad band gap originated from the vertical resonance. As a result, very effective frequency filter can be achieved if the rotational resonance is designed to be inside the band gap from the vertical resonance motion.

To check the feasibility of the frequency filtering, the metamaterial with the unit cell shown in Fig. 7(a) is numerically investigated. The unit cell in Fig. 7(a) is almost same as the previously considered unit cell in Fig. 5(a). However, the only difference is that the height of the inner mass is changed to 4 mm, which yields *m*_2_ = 0.002 kg and *I*_2_ = 2.709e − 09 kg⋅m^2^. The other geometric conditions, properties, and analytic coefficients are the same with the previous unit cell in Fig. 5(a) whose specifications can be found in the supplementary material. Figure 7(b) plots the numerically and analytically calculated wave dispersion curve. As can be inferred from the mode shapes plotted in Fig. 7(c), a narrow pass band near 89.3 Hz is originated from the rotational resonance, while the band gap from 48.73 to 117.65 Hz, originated from the vertical resonance. Thus, the metamaterial can be used to filter out the flexural waves from 48.73 to 117.65 Hz, except very narrow frequency range around 89.3 Hz.

**Figure 8:**
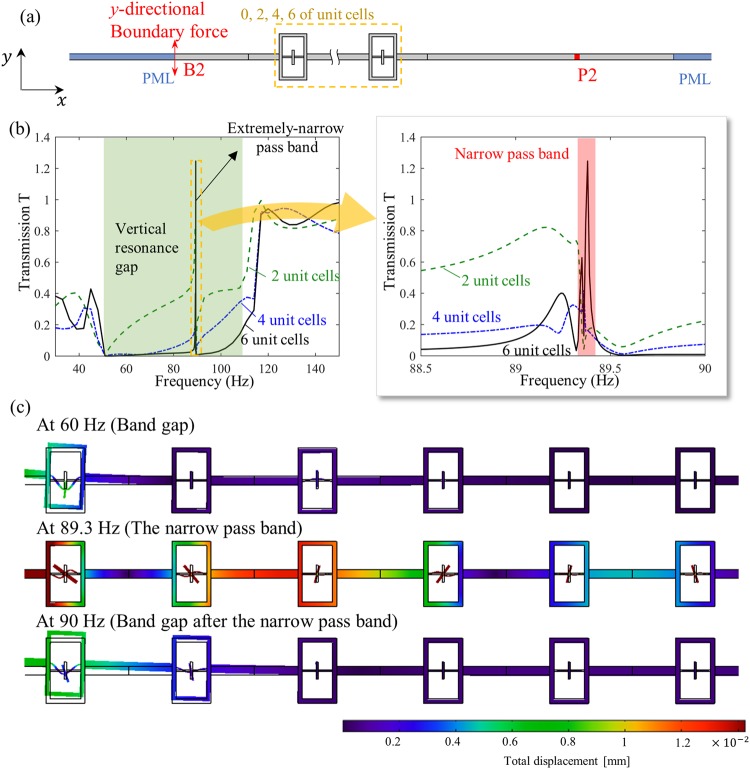
(**a**) Finite element modeling to measure the flexural wave transmission for the frequency filter, (**b**) transmission at various frequencies, (**c**) wave simulation results at various frequencies.

Figure 8(a) shows the wave simulation setting to check the performance of the frequency filtering with the unit cell shown in Fig. 7(a). Here, the same wave simulation procedure as in the previous section shown in Fig. 6(a) is used. Figure 8(b) shows numerically calculated wave transmission for various frequencies. As can be seen in Fig. 8(b), waves cannot propagate through the metamaterial layer at the frequency range from 50 to 100 Hz, except at 89.3 Hz. Figure 8(c) shows the deformed configuration of the metamaterial layer at the frequency of 60, 89.3, 90 Hz, respectively. In 60 and 90 Hz, the flexural waves cannot propagate through due to the vertical resonance motion of the first unit cell. However, at 89.3 Hz, the internal resonators exhibit rotational motion, and the flexural waves can propagate through by the help of the rotational motion. These results explain how the rotational resonance motion allows the wave propagation at very narrow frequency band. Thus, the metamaterial can filter out the waves at frequencies from 48.73 to 117.65 Hz, except the wave at frequency around 89.3 Hz, forming effective frequency filter.

## Discussion

In this paper, the abnormal band-gap phenomena in resonance-based flexural elastic metamaterials were reported and intensively studied. A new model dedicated on the resonance-based flexural elastic metamaterial was developed by extending Timoshenko theory. From the unique mass-spring system, the band gap condition and effective parameters were analytically investigated. The major findings from the analytic investigations can be summarized as follows; first, the vertical and rotational resonance independently affects the effective mass and effective rotation inertia, respectively. Second, the negative effective mass provides band gap, while the negative rotational inertia has nothing to do with the band gap. Third, the band gap originated by the vertical resonance is usually broad and includes the vertical resonance frequency, while the band gap generated by the rotational resonance is very narrow and formed outside the rotational resonance frequency. These findings were supported and validated with numerical simulations. It was shown that the findings can be generally applied for other cases where low frequency and pure mode assumptions can be used.

As the possible extension of the current findings, a frequency filtering device was introduced. From the analytic investigation, it was shown that a very narrow pass band can be formed inside a broad band gap if the resonator is well designed so that the rotational resonance frequency is inside the band gap of the vertical resonance motion. Simulations were carried out to show that only the flexural wave around a certain frequency can pass through the well-designed metamaterial layer. We expect our research can provide strong theoretical basis in the field of flexural elastic metamaterials that can open a new way in various vibration devices. Extending our research to high frequency ranges may also provide a new physics.

## Method

### Numerical dispersion curve simulation settings

The commercial finite element analysis program, COMSOL Multiphysics 5.3, is used. After modeling the unit cell, the wave dispersion curve is calculated by evaluating eigenfrequencies with the Floquet-Bloch condition imposed on the boundaries b1 and b2 in Figs 4(a) and 6(a)^36^. Note that since we are considering the one-dimensional mass-spring system, the unit cell has periodicity along *x*-direction only. Along *y*-direction, traction-free boundary conditions are considered.

### Transmission simulation settings

The simulation is carried out as follows. First, at the left edge B1 and B2, the *y*-directional harmonic force at various frequencies is applied. After that, the *y*-directional displacement w1 is measured at P1 and P2. For the comparison, the same analysis is repeated without the metamaterial layer and the *y-*directional displacement w2 is measured for each actuation frequencies. Finally, the transmission T is measured by the amplitude ratio of the measured *y-*directional displacement as T = |w1/w2|. In fact, the transmission T is not the exact definition of the transmission coefficient so that it may exceed 1. However, it can be effectively used to check the band gap formation. The same procedure was estimated for both unit cells array that this paper studied.

## Electronic supplementary material

Supplementary Material

## Data Availability

The datasets generated during and/or analyzed during the current study are available from the corresponding author on reasonable request.
