# Supplementary material for: Abnormal Stop Band Behavior Induced by Rotational Resonance in Flexural Metamaterial
Source: Sci Rep. 2018 Sep 24;8:14243. doi: 10.1038/s41598-018-32597-7 (PMC6155313; doi:10.1038/s41598-018-32597-7)
Supplement: Supplementary file 1 — Supplementary Material [file 41598_2018_32597_MOESM1_ESM.pdf]

Supplementary Materials

Abnormal Stop Band Behavior Induced by Rotational Resonance in Flexural Metamaterial

Sung Won Lee[1] and Joo Hwan Oh[1†]

[1] *School of Mechanical, Aerospace and Nuclear Engineering, Ulsan National Institute of Science and Technology, UNIST-gil 50, Eonyang-eup, Ulju-gun,Ulsan, 44919, Korea*

In this supplementary material, the detailed analytic procedures to derive the wave dispersion equations for the mass-spring systems shown in the manuscripts, and an analysis of abnormal behavior at rotational resonance are described. Also, detailed unit cell data, such as its material and geometric properties, are given.

[†] Corresponding Author, Email: joohwan.oh@unist.ac.kr, Tel: +82 52 217 3051

A. Analytic investigation for the discrete flexural metamaterials without inner resonators.

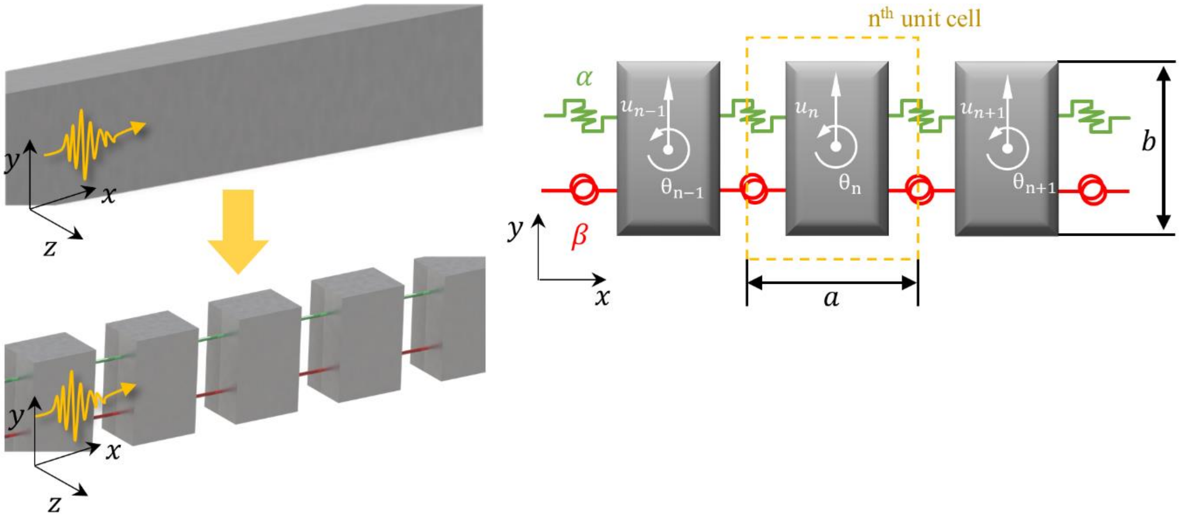

Figure S1. Equivalent mass-spring system for general flexural wave, two typical wave dispersion curves of flexural wave.

According to the classical Timoshenko theory [S1], the flexural wave can be viewed as a coupled dynamic behavior of the vertical and rotational motions. Thus, the mass-spring system used for longitudinal or shear wave cannot be used to describe the flexural wave. Here, we introduce the mass-spring system in Fig. S1 which can well-describe the general flexural wave [S2]. As can be seen in Fig. S1, each mass has two degrees of freedom, the vertical displacement u_n and the rotational displacement θ_n. Accordingly, two kinds of springs are used to connect each mass, the spring α that transfers the vertical force, and the rotational spring β that transfers the rotational moment. Note that the *x*-directional displacement is ignored since the *x*-directional motion is related to the longitudinal elastic wave, which is not covered by this paper.

First, based on the mass-spring system shown in Fig. S1, the wave dispersion equation for

flexural metamaterials without inner resonators can be analytically derived. As can be seen in Fig. S1, each mass has two degrees of freedom, the vertical displacement u_n and the rotational displacement θ_n. Accordingly, two kinds of springs are used to connect each mass, the spring α that transfers the vertical force, and the rotational spring β that transfers the rotational moment.

Here, a special attention should be paid for the rotational motion of each mass, θ_n. Due to the rotational motion θ_n, the vertical displacement of the nth mass is not same as u_n. Instead, the vertical displacement of the nth mass can be written as

$$\text{The left side of the n}^{\text{th}}\text{ mass: } u_n - \frac{a}{2}\theta_n, \tag{S1a}$$

$$\text{The right side of the n}^{\text{th}}\text{ mass: } u_n + \frac{a}{2}\theta_n. \tag{S1b}$$

Accordingly, the total vertical force acting on the nth mass is

$$\Sigma F_y = \alpha(u_{n+1} - \frac{a}{2}\theta_{n+1} - u_n - \frac{a}{2}\theta_n) + \alpha(u_{n-1} + \frac{a}{2}\theta_{n-1} - u_n + \frac{a}{2}\theta_n). \tag{S2}$$

Also, considering that the forces acting on the left and right side of the nth mass also generate moment, the total moment acting on the nth mass is

$$\begin{aligned}\Sigma M_o &= \beta(\theta_{n+1} - \theta_n) + \beta(\theta_{n-1} - \theta_n) \\ &\quad + \alpha\frac{a}{2}(u_{n+1} - \frac{a}{2}\theta_{n+1} - u_n - \frac{a}{2}\theta_n) - \alpha\frac{a}{2}(u_{n-1} + \frac{a}{2}\theta_{n-1} - u_n + \frac{a}{2}\theta_n)\end{aligned}. \tag{S3}$$

Since $\Sigma F_y = m\partial^2 u_n / \partial t^2$ and $\Sigma M_o = I\partial^2\theta_n / \partial t^2$, where m and I are the mass and the rotational inertia of the nth mass, Equations (S2, 3) can be re-arranged as

$$m\frac{\partial^2 u_n}{\partial t^2} = \alpha(u_{n+1} + u_{n-1} - 2u_n) + \alpha\frac{a}{2}(\theta_{n-1} - \theta_{n+1}), \tag{S4a}$$

$$I\frac{\partial^2 \theta_n}{\partial t^2} = \alpha\frac{a}{2}(u_{n+1} - u_{n-1}) + \beta(\theta_{n+1} + \theta_{n-1} - 2\theta_n) - \alpha\left(\frac{a}{2}\right)^2(\theta_{n+1} + \theta_{n-1} + 2\theta_n). \tag{S4b}$$

By Floquet-Bloch condition, u_{n+1} becomes $\exp(-ika)u_n$ and θ_{n+1} becomes $\exp(-ika)\theta_n$, while k is the wavevector. Assuming the time-harmonic solution with the angular frequency of ω, Equations (S4a, 4b) become:

$$-\omega^2 m u_n = \alpha\{\exp(ika)+\exp(-ika)-2\}u_n + \alpha\frac{a}{2}\{\exp(ika)-\exp(-ika)\}\theta_n, \tag{S5a}$$

$$\begin{aligned}-\omega^2 I\theta_n = \alpha\frac{a}{2}\{\exp(-ika)-\exp(ika)\}u_n + \beta\{\exp(ika)+\exp(-ika)-2\}\theta_n \\ -\alpha\left(\frac{a}{2}\right)^2\{\exp(ika)+\exp(-ika)+2\}\theta_n\end{aligned}. \tag{S5b}$$

Equations (S5a, 5b) can be more simplified by considering the Euler's formula as $\exp(ika)+\exp(-ika) \sim 2\cos(ka)$ and $\exp(ika)-\exp(-ika) \sim 2i\sin(ka)$. Substituting the Euler's formula yields

$$-\omega^2 m u_n = 2\alpha\{\cos(ka)-1\}u_n + i\alpha a\sin(ka)\theta_n, \tag{S6a}$$

$$-\omega^2 I\theta_n = -i\alpha a\sin(ka)u_n + 2\beta\{\cos(ka)-1\}\theta_n - \frac{\alpha a^2}{2}\{\cos(ka)+1\}\theta_n. \tag{S6b}$$

Finally, one can arrange the above equations in simple 2×2 matrix form as:

$$\begin{pmatrix} \omega^2 m + 2\alpha\{\cos(ka)-1\} & i\alpha a\sin(ka) \\ -i\alpha a\sin(ka) & \omega^2 I + 2\beta\{\cos(ka)-1\} - \frac{\alpha a^2}{2}\{\cos(ka)+1\} \end{pmatrix}\begin{pmatrix} u_n \\ \theta_n \end{pmatrix} = 0 \tag{S7}$$

which is the same equations with equations (1a, b) and (7) used in our main manuscript.

In fact, the above procedures are exactly same as the classical Timoshenko theory [S1]. To clarify this point, the spring and mass coefficients α, β, m, and I in Equations (S6, 7) are replaced by the equivalent coefficients in a continuum medium. For a bending beam, the spring coefficients are known as [S2]:

$$\alpha = \frac{GA\kappa}{a}, \qquad \beta = \frac{EI_b}{a} \tag{S8a, b}$$

where G, E, A and I_b is the shear modulus, Young's modulus, the cross-sectional area

and the cross-sectional momentum of inertia, respectively. Also, κ is the shear correction factor. In the same manner, m, and I can be written as;

$$m = \rho a A, \qquad I = \rho a I_b . \tag{S8c, d}$$

Note that for a continuum medium, the periodicity a is extremely small and it can be assumed that $\cos(ka) \sim 1-(ka)^2/2$ and $\sin(ka) \sim ka$. Substituting Equation (S8) and the assumptions to Equation (S7) yields

$$\begin{pmatrix} \rho A\omega^2 - GA\kappa k^2 & iGA\kappa k \\ -iGA\kappa k & \rho I_b\omega^2 - GA\kappa - EI_b k^2 \end{pmatrix} \begin{pmatrix} u_n \\ \theta_n \end{pmatrix} = 0 \tag{S9}$$

which is exactly same as the dispersion equation of the classical Timoshenko beam theory [S1]. Therefore, the analytic procedure carried out here is only valid for low frequency ranges. At high frequencies, the flexural wave should be considered as the anti-symmetric guided Lamb wave mode, which cannot be described with the proposed discrete system.

Also, from Equation (S8), it can be inferred that $\alpha a^2 > 4\beta$ generally holds for most cases. To explain this point, consider a flexural wave propagating in a beam with rectangular cross-section with width b and thickness h. In this case, the cross-sectional area is $A = bh$ and the cross-sectional momentum of inertia is $I_b = bh^3/12$. From Equation (S8), it can be calculated that

$$\alpha a^2 = Gbh\kappa a, \qquad 4\beta = Ebh^3/3a . \tag{S10a, b}$$

As seen in Equation (S10), αa^2 is proportional to the thickness h, while 4β is proportional to h^3; however, in general mechanical system where the flexural wave is mainly considered, the thickness h is usually very thin that $h << 1$. Therefore, αa^2 is usually several orders larger than 4β.

B. Analytic investigation for the discrete flexural metamaterials with inner resonators.

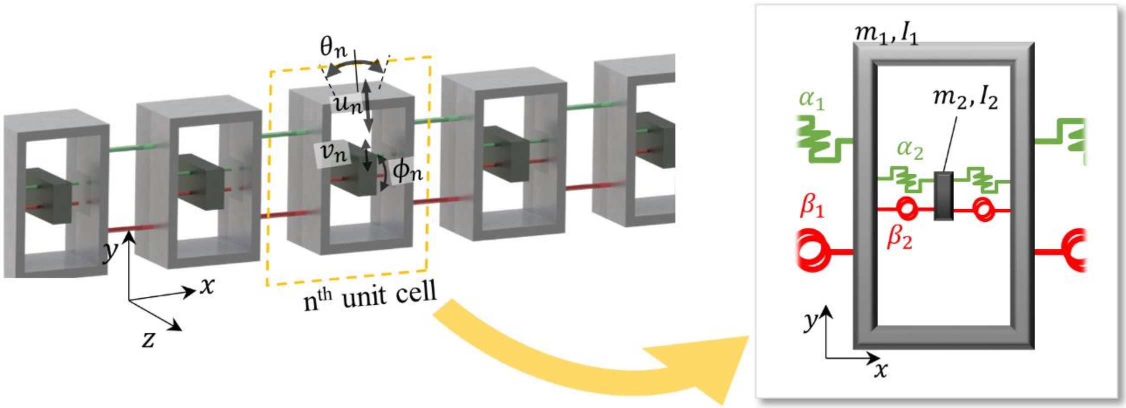

Figure S2. Equivalent mass-spring system for flexural elastic metamaterial with internal resonator.

Now, consider the mass-in-mass system for the flexural elastic wave shown in Fig. S2 which is equivalent to flexural elastic metamaterials with internal resonators. As in Fig. S1, both the vertical displacements (u_n, v_n) and rotational displacements (θ_n, ϕ_n) are considered for outer and inner masses. Thus, m_1 and I_1 correspond to the mass and rotational inertia of the outer mass, while m_2 and I_2 correspond to those of the inner mass. Also, the spring α_1 and rotational spring β_1 connect the outer mass of nth unit cell with the other mass of n+1th (or n-1th) unit cell, while the spring α_2 and β_2 connect the outer and inner masses of nth unit cell, as in Fig. S2.

Based on the mass-spring system, the wave dispersion equation is calculated. To this end, the effect of each mass' rotation, θ_n and ϕ_n, on its vertical displacements, u_n and v_n, should be considered first as in the previous approach. Assuming the length of the outer and inner mass is a_1 (which is same as the periodicity) and a_2 respectively, and assuming the length of all springs are zero, the vertical displacements of each mass are

The left side of the outer nth mass:

For the springs α_1 and β_1: $u_n - \frac{a_1}{2}\theta_n$ (S11a)

For the springs α_2 and β_2: $u_n - \frac{a_2}{2}\theta_n$ (S11b)

The right side of the outer nth mass:

For the springs α_1 and β_1: $u_n + \frac{a_1}{2}\theta_n$ (S11c)

For the springs α_2 and β_2: $u_n + \frac{a_2}{2}\theta_n$ (S11d)

The left side of the inner nth mass: $v_n - \frac{a_2}{2}\phi_n$ (S11e)

The right side of the inner nth mass: $v_n + \frac{a_2}{2}\phi_n$ (S11f)

Based on Equation (S11), the total forces and moments acting on each mass can be written as

For the outer nth mass:

$$\begin{aligned}\Sigma F_y = {} & \alpha_1(u_{n+1} - \frac{a_1}{2}\theta_{n+1} - u_n - \frac{a_1}{2}\theta_n) + \alpha_1(u_{n-1} + \frac{a_1}{2}\theta_{n-1} - u_n + \frac{a_1}{2}\theta_n) \\ & + \alpha_2(v_n + \frac{a_2}{2}\phi_n - u_n - \frac{a_2}{2}\theta_n) + \alpha_2(v_n - \frac{a_2}{2}\phi_n - u_n + \frac{a_2}{2}\theta_n)\end{aligned}, \tag{S12a}$$

$$\begin{aligned}\Sigma M_o = {} & \beta_1(\theta_{n+1} - \theta_n) + \beta_1(\theta_{n-1} - \theta_n) + \beta_2(\phi_n - \theta_n) + \beta_2(\phi_n - \theta_n) \\ & + \alpha_1\frac{a_1}{2}(u_{n+1} - \frac{a_1}{2}\theta_{n+1} - u_n - \frac{a_1}{2}\theta_n) - \alpha_1\frac{a_1}{2}(u_{n-1} + \frac{a_1}{2}\theta_{n-1} - u_n + \frac{a_1}{2}\theta_n) \\ & + \alpha_2\frac{a_2}{2}(v_n + \frac{a_2}{2}\phi_n - u_n - \frac{a_2}{2}\theta_n) - \alpha_2\frac{a_2}{2}(v_n - \frac{a_2}{2}\phi_n - u_n + \frac{a_2}{2}\theta_n)\end{aligned}. \tag{S12b}$$

For the inner nth mass:

$$\Sigma F_y = \alpha_2(u_n + \frac{a_2}{2}\theta_n - v_n - \frac{a_2}{2}\phi_n) + \alpha_2(u_n - \frac{a_2}{2}\theta_n - v_n + \frac{a_2}{2}\phi_n), \tag{S12c}$$

$$\begin{aligned}\Sigma M_o = {} & \beta_2(\theta_n - \phi_n) + \beta_2(\theta_n - \phi_n) \\ & + \alpha_2\frac{a_2}{2}(u_n + \frac{a_2}{2}\theta_n - v_n - \frac{a_2}{2}\phi_n) - \alpha_2\frac{a_2}{2}(u_n - \frac{a_2}{2}\theta_n - v_n + \frac{a_2}{2}\phi_n)\end{aligned}. \tag{S12d}$$

Reminding $\Sigma F_y = m\partial^2 u_n / \partial t^2$ and $\Sigma M_o = I\partial^2\theta_n / \partial t^2$, Equations (S12a-d) can be rearranged as

$$m_1 \frac{\partial^2 u_n}{\partial t^2} = \alpha_1 (u_{n+1} + u_{n-1} - 2u_n) + \alpha_1 \frac{a_1}{2} (\theta_{n-1} - \theta_{n+1}) + 2\alpha_2 (v_n - u_n), \tag{S13a}$$

$$\begin{aligned} I_1 \frac{\partial^2 \theta_n}{\partial t^2} &= \beta_1 (\theta_{n+1} + \theta_{n-1} - 2\theta_n) - \alpha_1 \left(\frac{a_1}{2}\right)^2 (\theta_{n-1} + \theta_{n+1} + 2\theta_n) \\ &\quad + (2\beta_2 + \alpha_2 a_2^2 / 2)(\phi_n - \theta_n) + \alpha_1 \frac{a_1}{2} (u_{n+1} - u_{n-1}) \end{aligned}, \tag{S13b}$$

$$m_2 \frac{\partial^2 v_n}{\partial t^2} = 2\alpha_2 (u_n - v_n), \tag{S13c}$$

$$I_2 \frac{\partial^2 \phi_n}{\partial t^2} = (2\beta_2 + \alpha_2 a_2^2 / 2)(\theta_n - \phi_n). \tag{S13d}$$

Again, by Floquet-Bloch condition, u_{n+1} becomes $\exp(-ika_1)u_n$ and θ_{n+1} becomes $\exp(-ika_1)\theta_n$, while k is the wavevector. Assuming the time-harmonic solution with the angular frequency of ω, Equation (S13) becomes:

$$\begin{aligned} -\omega^2 m_1 u_n &= \alpha_1 \{\exp(ika_1) + \exp(-ika_1) - 2\} u_n \\ &\quad + \alpha_1 \frac{a_1}{2} \{\exp(ika_1) - \exp(-ika_1)\} \theta_n + 2\alpha_2 (v_n - u_n) \end{aligned}, \tag{S14a}$$

$$\begin{aligned} -\omega^2 I_1 \theta_n &= \beta_1 \{\exp(ika_1) + \exp(-ika_1) - 2\} \theta_n - \alpha_1 \left(\frac{a_1}{2}\right)^2 \{\exp(ika_1) + \exp(-ika_1) + 2\} \theta_n \\ &\quad + (2\beta_2 + \alpha_2 a_2^2 / 2)(\phi_n - \theta_n) + \alpha_1 \frac{a_1}{2} \{\exp(-ika_1) - \exp(ika_1)\} u_n \end{aligned}, \tag{S14b}$$

$$-\omega^2 m_2 v_n = 2\alpha_2 (u_n - v_n), \tag{S14c}$$

$$-\omega^2 I_2 \phi_n = (2\beta_2 + \alpha_2 a_2^2 / 2)(\theta_n - \phi_n). \tag{S14d}$$

By arranging Equations (S14c, d), one can obtain the vertical and rotational displacement relation between the inner and outer masses as

$$v_n = \frac{2\alpha_2}{2\alpha_2 - \omega^2 m_2} u_n, \qquad \phi_n = \frac{2\beta_2 + \alpha_2 a_2^{\ 2}/2}{2\beta_2 + \alpha_2 a_2^{\ 2}/2 - \omega^2 I_2}\theta_n. \quad \text{(S15a, b)}$$

Substituting Equation (S15) to Equations (S14a, b) yields

$$\begin{aligned} -\omega^2 m_1 u_n = & \frac{2\alpha_2 m_2 \omega^2}{2\alpha_2 - \omega^2 m_2} u_n \\ & + \alpha_1\{\exp(ika_1) + \exp(-ika_1) - 2\}u_n + \alpha_1 \frac{a_1}{2}\{\exp(ika_1) - \exp(-ika_1)\}\theta_n \end{aligned}, \quad \text{(S16a)}$$

$$\begin{aligned} -\omega^2 I_1 \theta_n = & \frac{(2\beta_2 + \alpha_2 a_2^{\ 2}/2) I_2 \omega^2}{2\beta_2 + \alpha_2 a_2^{\ 2}/2 - \omega^2 I_2}\theta_n + \beta_1\{\exp(ika_1) + \exp(-ika_1) - 2\}\theta_n \\ & - \alpha_1 \left(\frac{a_1}{2}\right)^2 \{\exp(ika_1) + \exp(-ika_1) + 2\}\theta_n + \alpha_1 \frac{a_1}{2}\{\exp(-ika_1) - \exp(ika_1)\}u_n \end{aligned}. \quad \text{(S16b)}$$

Re-arranging Equations (S16a, b) by assembling the mass and inertia terms yields

$$\begin{aligned} -\omega^2 (m_1 + \frac{2\alpha_2 m_2}{2\alpha_2 - \omega^2 m_2}) u_n = & \alpha_1\{\exp(ika_1) + \exp(-ika_1) - 2\}u_n \\ & + \alpha_1 \frac{a_1}{2}\{\exp(ika_1) - \exp(-ika_1)\}\theta_n \end{aligned}, \quad \text{(S17a)}$$

$$\begin{aligned} -\omega^2 (I_1 + \frac{(2\beta_2 + \alpha_2 a_2^{\ 2}/2) I_2}{2\beta_2 + \alpha_2 a_2^{\ 2}/2 - \omega^2 I_2})\theta_n = & \alpha_1 \frac{a_1}{2}\{\exp(-ika_1) - \exp(ika_1)\}u_n \\ & - \alpha_1 \left(\frac{a_1}{2}\right)^2 \{\exp(ika_1) + \exp(-ika_1) + 2\}\theta_n \\ & + \beta_1\{\exp(ika_1) + \exp(-ika_1) - 2\}\theta_n \end{aligned}. \quad \text{(S17b)}$$

Considering $\exp(ika) + \exp(-ika) = 2\cos(ka)$ and $\exp(ika) - \exp(-ika) = 2i\sin(ka)$ from the Euler's formula, Equations (S19a, b) become

$$-\omega^2 (m_1 + \frac{2\alpha_2 m_2}{2\alpha_2 - \omega^2 m_2}) u_n = 2\alpha_1\{\cos(ka_1) - 1\}u_n + i\alpha_1 a_1 \sin(ka_1)\theta_n, \quad \text{(S18a)}$$

$$\begin{aligned} -\omega^2 (I_1 + \frac{(2\beta_2 + \alpha_2 a_2^{\ 2}/2) I_2}{2\beta_2 + \alpha_2 a_2^{\ 2}/2 - \omega^2 I_2})\theta_n = & -i\alpha_1 a_1 \sin(ka_1) u_n \\ & + 2\beta_1\{\cos(ka_1) - 1\}\theta_n - \frac{\alpha_1 a_1^{\ 2}}{2}\{\cos(ka_1) + 1\}\theta_n \end{aligned} \quad \text{(S18b)}$$

which is the same equations with Equations (5a, b) used in our main manuscript.

C. Physical explanation of abnormal phenomena at rotational resonance

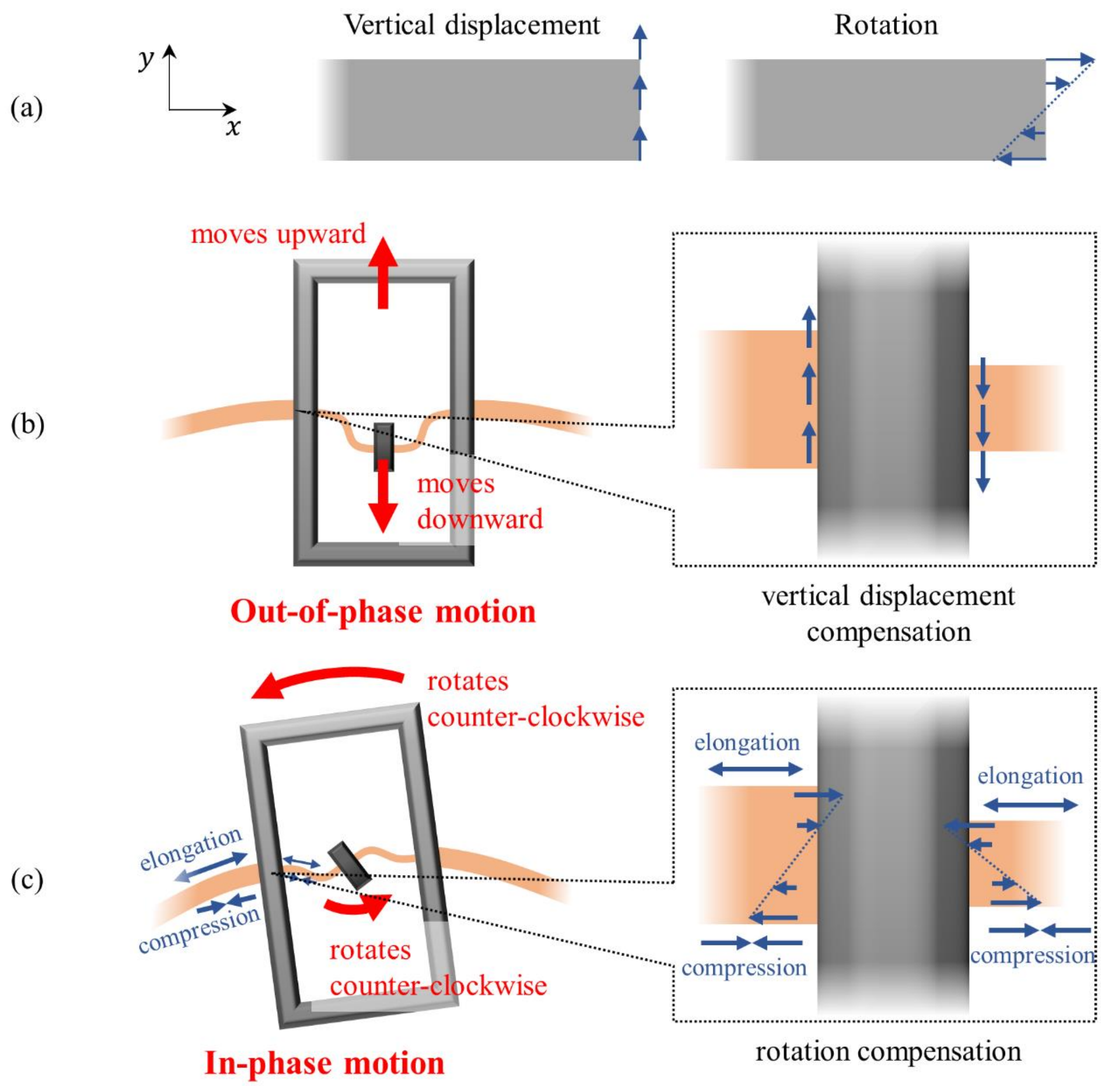

Figure S3. (a) Displacement profile for each deformation, band gap formation for (b) the vertical resonance and (c) rotational resonance

It is quite surprising that the negative rotational inertia does not generate any band gap. To more clearly show this point, the related phenomena is explained with the actual motion of the resonance based flexural metamaterial. Before the explanation, it should be noted that the vertical displacement and rotation can be described as the displacement profiles shown in Fig. S3 (a); the vertical displacement can be expressed as the uniform vertical motion along the

cross-section, while the rotation can be expressed as the combination of the positive and negative horizontal motions (or, the elongation and compression).

First, let us start from the well-known negative mass case shown in Fig. S3 (b). Assume that the incident wave forces the unit cell to move along the upward direction. To form the band gap, the internal resonator's motion should compensate this upward motion so that the unit cell would not move. Thus, the internal resonator should move downward – the out-of-phase motion is required. Considering that the negative mass is due to the large out-of-phase motion of the internal resonator, this explains why the negative mass generates band gap.

Now, assume that the incident wave forces the unit cell to rotate along the clockwise direction. This indicates that the upper part of the unit cell moves along the positive horizontal direction, while the lower part, moves along the negative horizontal direction. To compensate this motion, the internal resonator should exhibit elongation at the upper side and compression at the lower side, as shown in Fig. S3 (c). Therefore, the internal resonator should also rotate along the clockwise direction, i.e., the in-phase motion is required to form the band gap. This explains why the negative rotational inertia does not generate the band gap; the negative rotational inertia can be achieved when the out-of-phase motion takes place.

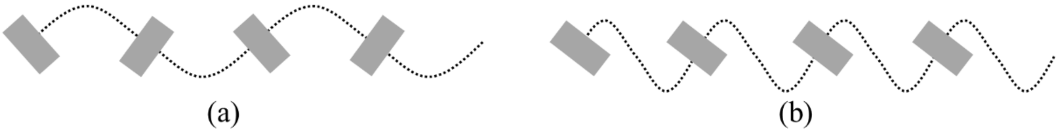

Figure S4. Rotational standing waves in periodic system. (a) Standing wave at the Bragg gap frequency, (b) standing wave at the cutoff frequency.

The reason why the infinite rotational inertia does not provide any Bragg gap is also

explained. At the condition of $I\omega^2 = 4\beta$, the periodic structure exhibit the rotational standing wave shown in Fig. S4 (a), forming the Bragg gap. Thus, if $I\omega^2$ is larger than 4β, stop band is formed due to the Bragg scattering. However, the periodic structure can also have another rotational standing wave shown in Fig. S4 (b) if $I\omega^2$ becomes αa^2. In fact, this is the well-known cutoff frequency of the high-order flexural wave branch - according to the classical Timoshenko beam theory, it has been known that there should be additional high-order branch starting from $\omega = \sqrt{\alpha a^2 / I}$. Thus, a new pass band is generated if $I\omega^2$ becomes larger than αa^2.

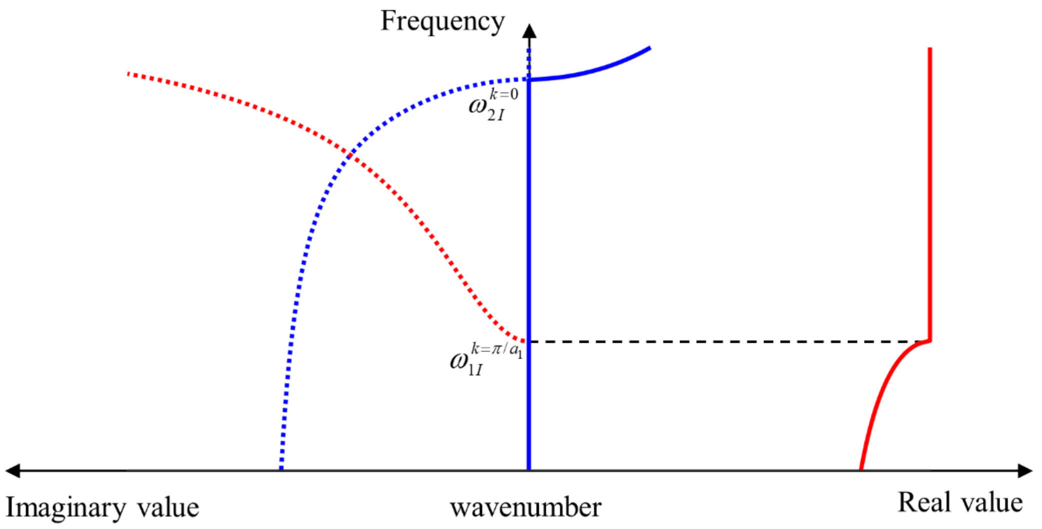

Figure S5. Plot of the wave dispersion curve around the second band gap.

The formation of the higher branch can be more clearly seen if the imaginary wavenumber is considered in the wave dispersion curve. Fig. S5 shows the wave dispersion curve with both the real and imaginary wavenumber at the frequency around the rotation-induced band gap. At the frequency of $\omega_{1I}^{k=\pi/a_1}$, it can be seen that the wave dispersion curve (red one in Fig. S5) goes to the imaginary plane, indicating that the Bragg gap is formed. On the other hand, one

can see that another wave dispersion curve (blue one in Fig. S5) enters to the real plane at the frequency of $\omega_{2I}^{k=0}$. Obviously, the blue and red branches are not the same one. The blue branch is the high-order branch explained in the Timoshenko beam theory, and this new branch is the main reason why the infinite rotational inertia does not provide Bragg gap.

D. Properties of the components for the numerical calculation

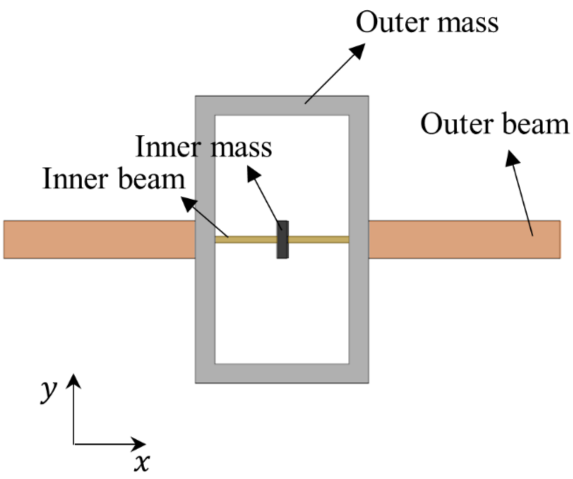

Figure S6. Unit cell of the metamaterial considered for the numerical investigations.

Fig. S6 shows the unit cell considered in the numerical investigations. The detailed geometric and material properties are given as below;

Outer mass: shell thickness 1 mm, width 9 mm, height 15 mm, density 10 kg/m^3,

Young's modulus 1000 MPa

Outer beam: width 10 mm, height 2 mm, density 0.2 kg/m^3, Young's modulus 1 MPa

Inner mass: width 0.5 mm, height 2 mm, density 1000 kg/m^3,

Young's modulus 10 MPa

Inner beam: width 3.25 mm, height 0.325 mm, density 0.0001 kg/m^3,

Young's modulus 0.1 MPa

It deserves to be noted that since our main goal is to verify the analytic investigations, artificial material properties are used. For instance, very large Young's modulus are used for

the outer and inner masses so that deformation can mainly takes place at the outer and inner beams, which are equivalent to the springs α_1, β_1, α_2, and β_2. Also, the densities of the outer and inner beams are set to be very small to minimize their inertia effects.

To compare with the numerically calculated dispersion curve, the dispersion curve is also analytically calculated with Equation (9). For the analytic calculation, the equivalent coefficients, such as m_1, I_1, α_1, and β_1, should be derived from the actual unit cell. These values can be calculated by using the classical vibration theory [S2]. For instance, the equivalent spring coefficients of the outer and inner beams can be calculated with Equations (S8a, b) from their static deformation. Using the data given above, the equivalent coefficients appearing in Equation (9) can be calculated as

$$m_1 = 4.4\times10^{-4}\,\text{kg},\ I_1 = 1.7983\times10^{-8}\ \text{kg}\cdot\text{m}^2,\ m_2 = 10^{-3}\,\text{kg},\ I_2 = 3.5417\times10^{-10}\,\text{kg}\cdot\text{m}^2 \quad \text{(S19a-d)}$$

$$\alpha_1 = 10^3\ \text{N/m},\ \beta_1 = 0.6667\,\text{N}\cdot\text{m/rad},\ \alpha_2 = 100\,\text{N/m},\ \beta_2 = 8.802\times10^{-5}\ \text{N}\cdot\text{m/rad} \quad \text{(S19e-h)}$$

$$a_1 = 0.029\,\text{m},\ a_2 = 0.0038\,\text{m} \quad \text{(S19i, j)}$$

With the parameters shown in Equation (S19), one can calculate m_{eff} and I_{eff} at any frequency from Equation (7), in our main manuscript.

References

[S1] K. F. Graff. Wave Motion in Elastic Solids. Dover Publications, Inc (2012).

[S2] M. Leonard. Fundamentals of vibrations. Mcgrow Hill (2001).

[S3] L. Brillouin. Wave propagation in periodic structures. Dover Publications, Inc (1946).
